# Knowledge and Risk Perception Regarding Keratinocyte Carcinoma in Lay People: A Systematic Review and Meta-Analysis

**DOI:** 10.3390/healthcare13151912

**Published:** 2025-08-06

**Authors:** Luisa Leonie Brokmeier, Laura Ilic, Sophia Haas, Wolfgang Uter, Markus Vincent Heppt, Olaf Gefeller, Isabelle Kaiser

**Affiliations:** 1 Department of Medical Informatics, Biometry and Epidemiology, Friedrich-Alexander-Universität Erlangen-Nürnberg, 91054 Erlangen, Germany; 2 Department Medical Psychology and Medical Sociology, Faculty of Medicine, Friedrich-Alexander-Universität Erlangen-Nürnberg, 91054 Erlangen, Germany; 3 Department of Dermatology, Uniklinikum Erlangen, Friedrich-Alexander-Universität Erlangen-Nürnberg (FAU), 91054 Erlangen, Germany; 4 Comprehensive Cancer Center Erlangen-European Metropolitan Area of Nürnberg (CCC ER-EMN), 91054 Erlangen, Germany; 5 Dermpath München, Laboratory for Dermatopathology, Oral Pathology and Molecular Pathology, 80335 Munich, Germany

**Keywords:** basal cell carcinoma, keratinocyte carcinoma, knowledge, risk perception, concern, prevention, skin cancer, squamous cell carcinoma

## Abstract

Background/Objectives: The increasing incidence rates of keratinocyte carcinoma (KC), particularly in fair-skinned populations, call for efforts to intensify health education of the general population in addressing this prevalent skin cancer type. As a preparatory step, this systematic review summarizes the published research on the knowledge and risk perception regarding KC among individuals without medical training. Methods: The review was registered in PROSPERO (CRD42024618851) and adheres to PRISMA guidelines. The databases PubMed, Scopus, Web of Science, PsycArticles, and PsycINFO were searched on 30 July 2024. Studies were eligible if knowledge and/or risk perception was assessed in lay people. Risk of bias (ROB) was assessed with the Joanna Briggs Institute checklist for prevalence studies. Comparable outcomes (e.g., awareness of terms for KC) were meta-analyzed. Results: Included reports (*n* = 17) were published between 1991 and 2024 with 16,728 individuals assessed. Awareness for the most common type of KC, basal cell carcinoma (BCC), was low (20.75% of respondents (95% confidence interval (CI): 15.24–27.61)), while more respondents were familiar with colloquial terms (60.9–72.8%). Meta-analysis indicated an underestimation of the frequency of KC, with only 7.21% (CI: 4.03–12.58) identifying BCC as the most common type of skin cancer. Furthermore, concern about developing KC as assessed in only two overlapping studies was reported by only 25–30% of respondents, indicating a significant gap in risk awareness and a lack of research on risk perception regarding KC. Conclusions: This review highlights the need for targeted health education interventions to improve knowledge and preventive behaviors regarding KC. Given the limitations of the included studies, characterized by high ROB, heterogeneity of results, and a lack of standardized assessment tools, further research is essential to enhance the understanding and awareness of KC in diverse populations.

## 1. Introduction

Knowledge about a disease and its prevention, as well as personal risk perception regarding the disease, are factors influencing preventive behavior [1,2]. This is of relevance especially for preventable diseases that are very common, such as several types of cancer [3]. One of the most common types of cancers—especially among fair-skinned populations—is skin cancer, more specifically keratinocyte carcinoma (KC) [4,5,6]. KC, often referred to as nonmelanoma skin cancer (NMSC), comprises basal cell carcinoma (BCC) and squamous cell carcinoma (SCC) [7]. The IARC Globocan database, comprising data from population-based cancer registries in 185 countries, shows that 1.23 million subjects were registered as incident KC cases in 2022 [8]. This reported incidence of KC seriously underestimates the global burden of these skin cancers, as some cancer registries do not routinely collect KC data and worldwide coverage of Globocan is incomplete [9,10]. Especially registration of BCC, which is the dominant subgroup of KC, is inadequate [11]. According to the Global Burden of Disease (GBD) Study [12], which employed sophisticated modeling techniques to correct for underreporting in registry data, there were an estimated 6.34 million incident cases of KC globally in 2021 [13]. Incidence rates are expected to increase further in the near future [6,14] and pose a large burden on healthcare systems [15]. Since for both BCC and SCC, extensive ultraviolet radiation (UV) exposure has been established as a strong risk factor [15,16], preventive measures in terms of a reduction in UV exposure could positively impact individuals’ health [17] and reduce the economic burden for healthcare systems [18]. A Belgian study conducted in 2016 estimated that investments in campaigns for skin cancer prevention would return 3.6 times of savings for healthcare providers [19].

Raising knowledge and awareness is an important preparatory step in the process of prevention. Even in secondary and tertiary prevention, health literacy is an important factor. According to a review, lower health literacy was associated with poorer quality of life and poorer experience of care in cancer patients [20]. Furthermore, trained medical personnel seem to overestimate health literacy in their (medically untrained) patients, impairing doctor–patient communication [21], highlighting the need to assess accurate levels of knowledge in lay people. As a starting point for prevention campaigns, it is therefore essential to first assess the knowledge and risk perception of KC among the general population to identify shortcomings. There are systematic reviews on skin cancer knowledge, e.g., [22] or consequences of sun exposure, e.g., [23], but not specifically for KC. A previous systematic review claims to have investigated knowledge and risk perception of KC [24]. However, none of the included studies assessed items on KC; they all referred to skin cancer in general. Nevertheless, the authors related their results to KC, since their goal was to provide advice on skin cancer prevention for outdoor workers. It is possible, though, that especially lay people with no professional training might perceive KC differently from skin cancer in general. Thus, to our knowledge, no systematic literature review has been conducted to address the knowledge and risk perception of KC in lay people. The aim of our work is to fill this gap by synthesizing all available information.

## 2. Materials and Methods

This systematic review was conducted and reported following the PRISMA guideline [25] (see Supplementary Material Table S1) as part of a larger review project registered with PROSPERO (registration number: CRD42024618851).

### 2.1. Eligibility Criteria

Following the SPIDER search strategy [26], all studies fulfill the following eligibility criteria:

- Sample: General population and subsamples thereof. Samples of participants with medical training (e.g., physicians, medical students, or nurses) or skin cancer patients were excluded.
- Phenomenon of Interest: Knowledge about, risk perception, or attitudes towards KC. This also includes awareness, familiarity, or beliefs. Studies focusing only on UV-related knowledge or UV behavioral assessment were excluded.
- Design: Cross-sectional surveys, cohort studies, baseline data of intervention studies.
- Evaluation: Items had to be sufficiently described to ascertain they were distinctly assessing the phenomenon of interest (i.e., no items referring to skin cancer in general or summary scores including other outcomes).
- Research Type: Quantitative, peer-reviewed studies. Qualitative studies, conference abstracts, dissertations, case reports, commentaries, editorials or reviews were excluded.

Studies published in German, English, and French were eligible.

### 2.2. Search Strategy

Five databases, including Medline (via PubMed), EMBASE (via Scopus), Web of Science, PsycArticles, and PsycINFO, were systematically searched using keywords and MeSH terms regarding knowledge and risk perception in combination with skin cancer (e.g., melanoma, nonmelanoma, keratinocyte carcinoma) and terms referring to the study design (e.g., interview* or cross-sectional). The detailed search strings and the syntax of the searches in the five databases can be found in the Supplementary Material Text S1. The search was conducted on 30 July 2024.

Furthermore, the method of forward and backward citation tracking [27,28] was used. For the latter, three systematic reviews [22,23,24] as well as one study [29] with similar research questions as this review were selected and their reference list extracted from Scopus. For the forward citation tracking, Scopus was used to identify and export all publications citing three main studies [30,31,32] on knowledge and risk perception regarding skin cancer. Before implementation and in accordance with the PRESS guideline [33], the search process had been reviewed by members of the team who were not involved in its original development.

All references rendered were allocated to an Endnote [34] library and duplicates were eliminated. The screening process was conducted and organized using the systematic review management software Rayyan [35]. In pairs, two researchers independently reviewed the titles and abstracts of this reference list for relevant articles (first screening phase). Following the rationale of sensitivity, inclusion by only one reviewer was sufficient to include the article for the full-text screening. In this second screening phase, the entire article was read by two researchers independently to decide upon its eligibility. During this screening phase, labels were applied to identify studies with items referring to knowledge and risk perception regarding KC. Additionally, all full texts were searched for KC-related terms to ensure that no studies were missed. Disagreements were discussed until a consensus was reached. If uncertainties prevailed, a third researcher was consulted.

### 2.3. Data Extraction

Two researchers independently extracted data on sample characteristics, item specifics, and results for each study using the Agency for Healthcare Research and Quality’s (AHRQ) Systematic Review Data Repository Plus (SRDR+, https://srdrplus.ahrq.gov, last accessed on 30 April 2025). Study and sample characteristics were collected on country and date of data collection, population, recruitment procedure, sample size, percentage of female and male participants, and participants’ age. As outcomes, we extracted details on the exact wording of the items assessed. If this was not explicitly stated in the methods section or in the Supplementary Material, the phrase used in the studies to report their results was extracted. The number of analyzed participants, number and/or percentage of responses, as well as further outcome details (e.g., sex differences) were extracted. On SRDR+, a form was created to ensure a standardized extraction process. We piloted the extraction with two randomly selected studies to detect any misunderstandings. SRDR+ automatically detects conflicts between extractions, which were checked and resolved by an independent third researcher.

### 2.4. Risk of Bias (ROB) Assessment

To assess the ROB of the individual studies, the Joanna Briggs Institute (JBI) checklist for prevalence studies [36] was applied. It includes nine items relating to sample selection, outcome measurement, analysis methods, and reporting standards. One item specifically assesses whether the sample size of the study is sufficient and thus requires a decision, in which a minimum sample size is considered sufficient. We set *n* = 250 as the required minimum sample size. This is to some extent arbitrary but justified as studies with a smaller sample size lead to estimates of proportions with 95% confidence intervals (CIs) that can exceed 10% in width, which we consider to be too wide. If criteria were met, items were answered with yes (versus no). Too little information reported in the study led to the verdict ‘unclear’. The JBI tool does not provide detailed instructions on how to conclude an overall ROB. We based the overall ROB rating on a joint critical appraisal of all relevant aspects, with low methodological study quality in key aspects not being compensated for by other components of the study with a higher methodological quality. We therefore established a rating standard as follows: High concerns or an unclear rating regarding items indicating a selection bias, measurement bias, or an inadequate sample size led to an overall rating of high ROB or unclear, respectively. In particular, inadequate sampling strategies and response rate management, invalid methods of measurement, or sample sizes below 250 were rated as high ROB. After establishing these decision criteria, studies were rated independently by two researchers each. In the case of discrepant ROB ratings, consensus meetings were held to discuss the disagreements and reach a consensus decision.

### 2.5. Data Synthesis

The main characteristics and ROB ratings of the included studies were summarized in a table. The results (i.e., proportions) regarding knowledge and risk perception were classified into subcategories to compare outcomes. Outcomes were comparable, if the items assessed the same construct on the same scale, reporting quantitative data on the proportion of the sample in said outcome categories. As subcategories for KC-specific knowledge, four topics were identified: (1) awareness of terms for KC, (2) identification of KC as a type of skin cancer, (3) knowledge regarding the prevalence of KC, and (4) more detailed, specific knowledge regarding KC. There was no study reporting risk perception regarding KC. However, concern about developing KC was found in two studies. Meta-analyses were conducted for comparable items if at least three studies were allocated in a subgroup. If not provided by the study, proportions and their corresponding confidence intervals (CIs) were calculated, using the Wilson method for the latter. The meta-analyses were conducted using a generalized linear mixed model (GLMM) with random effects. Between-study heterogeneity was assessed by calculating the *I*^2^-statistic (range 0–100%, with high heterogeneity assumed for *I*^2^ ≥ 75%) [37]. The maximum-likelihood estimator of the between-study variance (*τ*^2^) was derived from the GLMM and is additionally reported. In a scenario with high between-study heterogeneity, the 95% CI of the pooled estimate of the proportion of interest does not adequately reflect the uncertainty of statements about that proportion [38]. Therefore, we expanded the forest plot to also show the 95% prediction interval (PI) of the proportion of interest [39]. A 95% PI is defined as a range for the true proportion of interest that covers 95% of future study results. In contrast to the CI for the pooled meta-analytical estimate, the PI incorporates the structural between-study heterogeneity. We did not conduct statistical tests for publication bias since it is advised not to use them when pooling proportions [40]. Analyses were conducted in R version 4.2.2 using the R packages ‘meta’ and ‘metafor’.

## 3. Results

**Figure 1:**
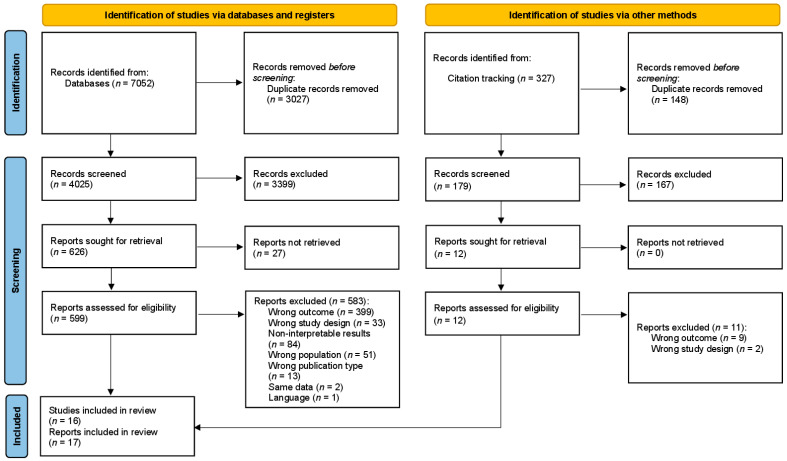
PRISMA flowchart of the systematic literature review process.

The search rendered initially 7379 references. After removal of duplicates, 4204 studies were screened in the first phase, and 638 in the second. Knowledge of and attitudes towards KC as defined in our inclusion criteria were addressed in 17 studies (see Figure 1; for a detailed list of exclusions during the full text screening, see Supplementary Table S2). Three studies [41,42,43] were excluded even though they explicitly addressed KC knowledge and risk awareness in their titles and/or abstracts. However, the items reported were only referring to ‘skin cancer’ in general, thus not meeting the predefined inclusion criteria. One study [44] was identified because it was cited in the methods section of one of the included studies [45]. Two of the included studies analyzed overlapping data from the same study [46,47]. Therefore, only data on the target population of outdoor workers was extracted from the second study [47].

Included studies were published between 1991 and 2024, with data assessed in Europe, USA, Australia, and Saudi Arabia. Sample sizes ranged from 37 to 4000, summing up to 16,728 individuals assessed (corrected for overlap between [46,47]). Age of participants ranged from 14 to 89 years, with proportions of female participants ranging from 0% to 70%. ROB was rated low in two studies, high in eight studies, and unclear in seven studies (Table 1). Inadequate convenience sampling or small sample sizes were the main reasons for high ROB ratings. Unclear ratings were due to insufficient reporting of study details.

### 3.1. Awareness of Terms for KC

Seven studies investigated whether participants were familiar with or had heard of terms referring to KC (Table 2). Colloquial terms for KC were assessed in two studies [46,47] and were most familiar to participants (60.9–72.8%). Awareness regarding the medical terms BCC (9.8–30.7%) [30,46,47,49,50,54,55] and SCC (22.6–23.0%) [46,47] was lower.

**Figure 2:**
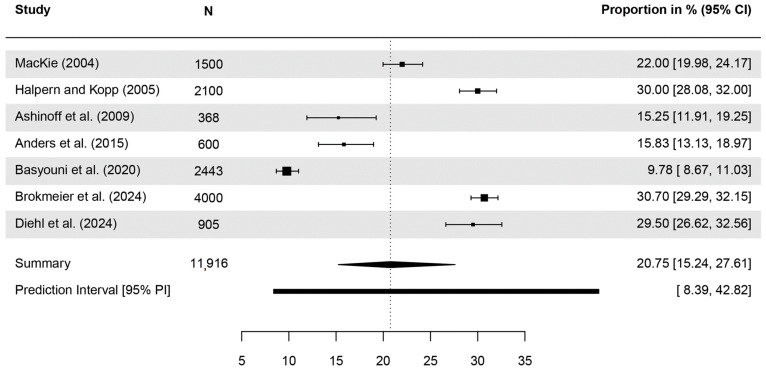
Forest plot with meta-analysis showing the proportions of the awareness of the term basal cell carcinoma (BCC). CI: Confidence interval [30,46,47,49,50,54,55].

A meta-analysis of seven studies reporting quantitative data on awareness of the term BCC indicated a pooled estimate of 20.75% (95% CI = 15.24–27.61, *τ*^2^ = 0.25, *I*^2^ = 98.6%, *p* < 0.001; Figure 2). In one study, 32.7% of the sample knew at least one of four terms referring to SCC, while 12.8% knew of the term ‘Basaliom’ (German for BCC) [54]. Two studies [46,54] reported gender differences with females reporting a higher level of awareness of KC-specific terms.

### 3.2. Identification of KC as a Type of Skin Cancer

**Figure 3:**
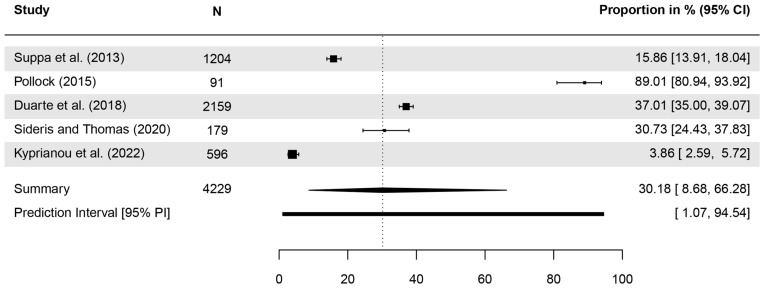
Forest plot with meta-analysis showing the proportions of the identification of basal cell carcinoma (BCC) as a type of cancer. CI: Confidence interval [29,44,45,52,58].

**Figure 4:**
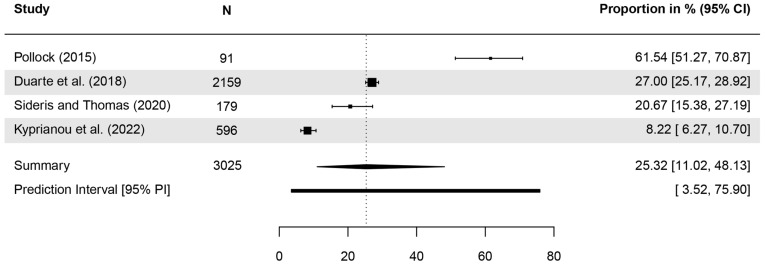
Forest plot with meta-analysis showing the proportions of the identification of squamous cell carcinoma (SCC) as a type of cancer. CI: Confidence interval [29,44,45,58].

In six studies, participants were asked to identify BCC and SCC as types of cancer. Correct answers ranged from <10% [58] to 90% [44] (Table 3). Meta-analyses indicated that 30.18% (CI = 8.68–66.29, *τ*^2^ = 2.95, *I*^2^ = 98.9%, *p* < 0.001, included studies [k] = 5; Figure 3) and 25.32% (CI = 11.02–48.13, *τ*^2^ = 1.03, *I*^2^ = 97.8%, *p* < 0.001, k = 4; Figure 4) of respondents were able to identify BCC and SCC, respectively.

### 3.3. Knowledge Regarding the Prevalence of KC

**Figure 5:**
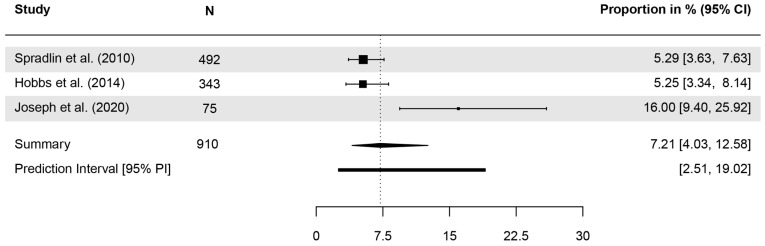
Forest plot with meta-analysis showing the proportions of the identification of basal cell carcinoma (BCC) as the most common type of cancer. CI: Confidence interval [51,53,57].

Three studies investigated whether participants knew that BCC is the most common type of skin cancer. The correct response rate ranged from 5.2 to 16%, with a pooled proportion of 7.21% (CI = 4.03–12.58, *τ*^2^ = 0.22, *I*^2^ = 83.7%, *p* = 0.002, k = 3; Figure 5). When asked to estimate the prevalence of KC in comparison to malignant melanoma [46], only 27.4% correctly assumed KC to be more prevalent (Table 4).

### 3.4. Specific Knowledge Regarding KC

Four studies investigated specific knowledge regarding KC (Table 5). Correct responses regarding signs of KC ranged from 18.0% (‘Light or white spots on the skin’) to 72.8% (‘Reddish, rough, scaly skin spots’) [46,47]. Identifying which was not a sign of BCC was incorrectly answered by 41% [48]. Sunlight was known as a factor contributing to KC by 71.4–78.7% [46,47]. Almost half of the sample of the general population overestimated the severity of KC compared to malignant melanoma (equally severe: 37.4%, more severe: 12.4%), while 21.2% answered ‘I don’t know’ [46]. In another study, 43–73% of the participants were able to identify BCC and SCC as malignant in pictures of skin lesions [56]. Specific facts about BCC and SCC were asked as multiple-choice items in one study and yielded incorrect responses of 86% (SCC) and 52% (BCC), respectively [48].

### 3.5. Concern About Developing KC

Two studies [46,47] reported whether participants were concerned about developing KC. A greater proportion of the sample of outdoor workers expressed their concern (30.0%) compared to the general population (25.0%). However, in both samples, an even larger part of the study participants (32.0–37.3%) had never thought about it (Table 6).

## 4. Discussion

Our search for studies investigating knowledge and risk perception regarding KC identified 17 eligible studies for inclusion in this systematic review. Only two of these studies were rated as low in ROB, indicating a lack of high-quality research on the topic. All studies were conducted in countries of the Northern Hemisphere and Australia. Concerning South America, Africa, and Asia with their partly fair-skinned populations and non-negligible NMSC mortality rates [59], respectively, further research into relevant knowledge domains seems warranted. It is worrisome that there are no studies in these regions, and thus no indication of what knowledge level to build prevention campaigns on. This will lead to even greater inequalities in the future. While skin cancer incidence is expected to rise globally, this increase is predicted to be stronger in low- and middle-income countries [60]. In these countries, healthcare infrastructure and financial budgets for prevention are insufficient, which exacerbates existing inequalities [60]. The instruments used in the studies to assess knowledge and risk perception, as well as the sociodemographic and constitutional characteristics of the study samples, showed vast heterogeneity.

Overall, our synthesis of the study results revealed a low level of knowledge and awareness of KC: Only slightly more than one-fifth of respondents had heard of BCC or SCC. Acknowledging the fact that BCC and SCC are medical terms, the awareness of colloquial terms for KC was substantially higher in one German study. Still, about 30–40% of lay people were unaware of KC. In comparison, the proportions of individuals having heard of skin cancer are far higher (e.g., 97%) [52]. There is clearly a lack of awareness of the differentiation of skin cancer into different types, leaving the two most common types, BCC and SCC, unknown to most lay people. In women, this deficit of awareness seems to be less pronounced: Two of the included studies reported sex differences in awareness [46,54], with women being more aware of KC-related terms. This is in line with previous research, showing that women seem to be more knowledgeable of health topics in general [61]. The authors argue that this might be due to women’s role as care takers in families or because they might have more experience in navigating the healthcare system. Therefore, it might be reasonable to focus health education primarily on men [62]. Unfortunately, only two studies reported gender differences, but one of these did not report actual gender-specific proportions for women and men separately [54]. More research should determine whether there is a persistent gender knowledge gap.

Furthermore, the disease frequency of KC is underestimated by lay people: Only one in fourteen could identify BCC as the most common type of skin cancer. In the one study reporting all answers given, melanoma was assumed to be the most common form (63%) [57]. This might be due to the fact that melanoma is more fatal than KC, and thus receives more attention in health education campaigns [63]. However, since KC is more prevalent and incidences are expected to increase [64], it deserves more awareness.

The fact that UV radiation is a major risk factor for KC seems to be more common knowledge (71.4–78.7%). However, awareness of this causal relationship with skin cancer in general as opposed to KC seems to be far higher, reaching proportions of more than 90% [24]. This may indicate that some individuals regard KC as an entirely distinct form of cancer. On the other hand, one could argue that the proportion of awareness about the causal relationship between UV radiation and KC is surprisingly high, given the fact that most respondents in our analyses were unaware of KC. This might be explained by language differences: UV radiation as a risk factor for KC was an item in the German study [46,47], and was assessed using the colloquial German term for KC (‘white skin cancer’). This might have encouraged respondents to mingle in their knowledge about skin cancer in general or it might indicate that respondents conflate risk factors for KC and melanoma. This hypothesis is further supported by answers to items investigating knowledge about signs of KC. In Brokmeier et al. [46], more than 20% of respondents stated that changes in nevi are signs of KC, indicating that these respondents do not distinguish between KC and melanoma. Thus, there is conflicting evidence from the studies as to whether KC is perceived as a distinct type of skin cancer, or whether it is considered the same as or even a subtype of melanoma. Future research should investigate what exactly it is that the general population considers to be KC. This might have further implications for health education. Possibly, it would need to address a basic understanding of BCC and SCC as distinct types of skin cancer before conveying any more detailed information. All types of skin cancer are linked to UV exposure, but the harmful effects of different UV exposure patterns vary depending on the skin cancer type [65]. Populations with different sun exposure patterns thus require different skin cancer education specifically tailored to their needs, which means considering the pre-intervention knowledge level of the targeted population [66]. For instance, chronic UV exposure increases especially the risk for SCC, making this type of skin cancer highly relevant for outdoor workers [67]. Furthermore, signs vary among different types of skin cancer. Therefore, to promote secondary prevention, such tailored health education could include information on what signs to look for depending on skin cancer type. Even though the German Guideline Program in Oncology (GGPO) on Skin Cancer Prevention [68] highlights the importance on secondary prevention, it focuses only on melanoma in this regard. Our findings indicate that additional focus on the early detection of KC is needed. Acquisition of knowledge on risk factors and prevention should best be based on complete and reliable sources of information, such as the GGPO. However, it has been shown that a minority of internet resources meet these prerequisites [69].

Knowledge and risk perception are both factors included in several health behavior models (e.g., Behavior Change Wheel [70]; Health Belief Model [71]; Health Action Process Approach [72]). According to these models, knowledge alone is insufficient to prompt individuals to engage in preventive behavior. Beliefs or perceptions that there is a risk to the individual’s health play an essential role in the process of health behavior change [73]. For instance, a systematic review found associations between risk perception regarding breast cancer and preventive behavior [74]. As recommended by Edmonds et al. [75], risk perception regarding cancer should be assessed as comparative risk, i.e., asking participants to estimate their risk compared to others. However, our systematic search identified only two studies [46,47], using overlapping data, which investigated a form of risk perception about KC, namely, concern about developing KC. Results of these studies suggest that about one third of interviewed individuals have never thought about developing KC as opposed to a quarter of respondents actually being concerned, while the rest had never thought about it. This, as well as the underestimation of the high incidence of KC, indicate that there is a lack of awareness regarding the high probability of developing KC during lifetime, which, for example, has reached 69% in Australia [76]. Supporting this, a low risk awareness regarding KC was found in qualitative studies [77,78]. However, there is obviously a lack of quantitative data to draw public health policy conclusions. The high prevalence of KC calls for further studies addressing the perception of its risk in the general population.

Even though we did a comprehensive search for studies investigating knowledge and risk perception of KC with search terms covering a broad range of possible concepts and research items, we cannot exclude having missed relevant studies. Furthermore, as outlined in the Methods section, a formal assessment of publication bias for non-significant epidemiological or survey data is not recommended. However, we would expect a publication bias in this line of research favoring large sample sizes. Yet, we have found and included four studies with less than 250 participants, suggesting a probably negligible bias. The low number of studies included did not allow for quantitative syntheses of results for all subcategories of outcomes, let alone sensitivity analysis focusing exclusively on studies of low ROB or other subgroup analyses. A further problem that impedes the comparability of results is the considerable measurement heterogeneity in the studies. The resulting high heterogeneity of study-specific results is further reflected in very broad prediction intervals, indicating that future studies may find proportions for percentages of people aware of BCC ranging between one and 95%, in one instance. In particular, KC-specific knowledge items were too heterogeneously assessed in the included studies to be reasonably compared with another. For such a comparison, a standardized instrument would be required. Some studies, e.g., [79], have used the Skin Cancer and Sun Knowledge (SCSK) Scale [80] which assesses knowledge about sun protective behavior and (fewer) items on skin cancer in general. Two items of 25 refer to both melanoma and KC (most common type of skin cancer and signs of skin cancer). However, KC is not stated specifically. KC as the most common type of skin cancer deserves a more prominent role in the assessment of skin cancer knowledge. The SCSK could be adapted and used as a standardized assessment tool. Future items could assess knowledge about risk factors for and consequences of KC, types of skin cancer, or focus on the high prevalence of KC. In addition to a standardized assessment of KC-specific knowledge, transparent and complete reporting in the publications is also essential. This approach is imperative for the accurate interpretation of results and the evaluation of study quality. The STROBE guidelines [81] have been developed for the reporting of observational studies. It is recommended that subsequent studies adhere to these guidelines. Furthermore, analyses of temporal changes in knowledge levels over the last decades, which have been performed in the context of knowledge about melanoma risk factors [32,82], would be interesting but would require more comparable outcomes, as well. In two German reports [46,47], a culture-specific colloquial term (translated as ‘white skin cancer’) was used for KC in assessing knowledge. Proportions in awareness for colloquial terms might have been higher in other populations, but this has not been explored in other studies. This calls for further research in other cultures with their corresponding colloquial KC terms. Overall, the lack of high-quality research as well as the high heterogeneity observed in the conducted meta-analyses mandates caution in interpreting or generalizing the reported results. To decrease ROB, future studies should adhere diligently to adequate sampling strategies, since this was the main reason for high ROB ratings.

## 5. Conclusions

To our knowledge, this is the first systematic review of published literature on knowledge about and perception of KC. Overall, knowledge of and concern about KC seem to be rather low. However, only few studies were found, especially addressing the perception of KC, and those found showed heterogenous results and mostly a high ROB. Synthesizing the evidence from these studies was additionally hampered by the lack of standardized instruments for assessing outcomes related to knowledge and perception. This calls for the development of standardized assessment instruments and more research in this area, ultimately leading to better health education on KC for the general population.

## Figures and Tables

**Table 1:** Study characteristics of all included publications, ordered by publication date.

Publication	Country	Assessment Date	Population	Method of Recruitment and Information Assessment	Sample Size (% Female)	Age (Years)	ROB
Katz, Jernigan (1991) [48]	USA	n.r. **	Undergraduate college students	Sample of college students from various undergraduate classes at a small private liberal arts college (unclear whether full census, convenience or random sample); self-administered questionnaire	251 (n.r.)	Range: 16–35	High
MacKie (2004) [30]	UK, France, Italy, Germany, Spain	n.r. *	General population	Selected randomly from telephone directories, in-house databases and random dialing; 10 min structured telephone interview	1500 (40%)	Range: 40–70	Unclear
Halpern, Kopp (2005) [49]	UK, France, Italy, Germany, Spain, USA, Australia	n.r. *	General population	Selected randomly from telephone directories, in-house databases and random dialing (market research survey); 10 min structured telephone interview	2100 (40%)	Range: 40–70	Unclear
Ashinoff et al. (2009) [50]	USA	n.r. *	High school students in grades 9 through 12	Voluntary, anonymous survey administered to more than 450 high school students in two schools; self-administered questionnaire	368 (‘roughly 50%’)	Mean: 16 Range: 14–18	Unclear
Spradlin et al. (2010) [51]	USA	n.r. *	Undergraduate students at a mid-sized southern university	Participants obtained through lecture hall classes across campus; self-administered questionnaire	492 (51.4%)	Range: 18–24	Unclear
Suppa et al. (2013) [52]	Italy	n.r. *	Students from 11 secondary schools	Randomly selected throughout the Abruzzo region in Central Italy; self-administered questionnaire	1204 (51.7%)	Median: 17; Range: 14–21 <18: *n* = 748 (62.1%) ≥18: *n* = 456 (37.9%)	Unclear
Hobbs et al. (2014) [53]	USA	n.r. *	Athletes from the Southern University in the USA	Convenience sample; self-administered questionnaire	343 (45.2%)	18: 10.4%; 19: 28%; 20: 22.3%; 21: 17.8%; 22: 16.9%; 23: 3.2%; 24: 0.3%	High
Anders et al. (2015) [54]	Germany	April/May 2003 **	General population	Households contacted by phone by applying a random digit dialing algorithm; computer-assisted telephone interviews	600 (55.0%)	Mean: 49.7 ± 17.06; 20–29: *n* = 79 (13.2%) 30–39: *n* = 122 (20.4%) 40–49: *n* = 108 (18.0%) 50–59: *n* = 90 (15.0%) 60–69: *n* = 103 (17.1%) ≥70: *n* = 98 (16.3%)	Low
Pollock (2015) [44]	Australia	December 2012–February 2013 *	Patients from two medical practices in the Northern Rivers region	Participants recruited through information posters placed in the waiting rooms; self-administered questionnaire	91 (57.14% ***)	18–20: *n* = 10 21–24: *n* = 7 25–34: *n* = 13 35–44: *n* = 6 45–54: *n* = 17 ≥55: *n* = 38	High
Duarte et al. (2018) [29]	Spain and Portugal	November 2014–September 2015 *	Outdoor runners	All athletes registered for four consecutive races; self-administered online questionnaire	2445 (20%)	<25: *n* = 123 25–44: *n* = 1610 ≥45: *n* = 413	High
Basyouni et al. (2020) [55]	Saudi Arabia	2018 *	Residents of Jeddah (General population)	General population of Jeddah in multiple locations and different districts of the city (no further details); interviews and self-administered questionnaire	2443 (68.2%)	18–34: 73.6% 35–49: 16.1% ≥50: 10.4%	Unclear
Garcia et al. (2020) [56]	USA	n.r. **	Primary Spanish-speaking patients at a family medicine clinic	Convenience sample; self-administered questionnaire	37 (70%)	Mean: 50.92 ± 10.68	High
Joseph et al. (2020) [57]	USA	n.r. *	Homeless men	Convenience sample in a 335-bed, male only, homeless shelter; either self-administered or researcher-administered questionnaire, depending on the participant’s preference or ability to complete the survey	75 (0%)	18–44: *n* = 34 (45%) ≥45: *n* = 41 (55%)	High
Sideris, Thomas (2020) [45]	Australia	October 2015 *	Patients of a medical practice	Distribution of a voluntary paper-based questionnaire to all patients aged 18+; self-administered questionnaire	179 (62.57%***)	Range: 18–89 18–30: 15.64% 31–45: 23.46% 46–60: 29.05% 61–75: 20.11% >75: 10.61% Missing: 1.12%	High
Kyprianou et al. (2022) [58]	Cyprus	October 2015–April 2016 *	Cypriot residents	Participants recruited in public areas based on proportional quota sampling (no further details); either interviews or self-administered questionnaire	600 (53.0%)	18–24: *n* = 85 (14.0%) 25–29: *n* = 78 (13.0%) 30–34: *n* = 59 (10.0%) 35–39: *n* = 67 (11.0%) 40–44: *n* = 49 (8.0%) 45–49: *n* = 51 (9.0%) 50–54: *n* = 56 (9.0%) 55–59: *n* = 36 (6.0%) ≥60: *n* = 118 (20.0%)	High
Brokmeier et al. (2024) [46]	Germany	October–December 2022 *	General population	Participants selected using a two-stage random sampling procedure; standardized computer-assisted telephone interviews	4000 (49.3%)	Range: 16–65 Mean: 42.43 ± 14.02	Low
Diehl et al. (2024) [47]	Germany	October–December 2022 *	Former/current outdoor workers	Participants selected using a two-stage random sampling procedure; standardized computer-assisted telephone interviews	905 (36.2%)	16–25: *n* = 196 (21.7%) 26–35: *n* = 250 (27.7%) 36–45: *n* = 174 (19.2%) 46–55: *n* = 144 (15.9%) 56–65: *n* = 140 (15.5%)	Unclear

n.r.: not reported; ROB: Risk of bias. * Cross-sectional study. ** Baseline data of an intervention study. *** Calculated from information provided in the study.

**Table 2:** Awareness of terms for KC, ordered by publication date.

Publication	Awareness of the Term	N Analyzed	*n* (%) Aware	Further Details
MacKie (2004) [30]	BCC	1500	22%	
Halpern, Kopp (2005) [49]	BCC	2100	30%	
Ashinoff et al. (2009) [50]	BCC	368	15.25% *	‘…almost 85 percent were not familiar with a basal cell carcinoma’
Anders et al. (2015) [54]	At least one of four terms referring to SCC **	600	194 (32.7%)	‘Women knew more often all terms for skin cancer compared with men (*p* < 0.002)’
	‘Basaliom’	600	76 (12.8%)	
	BCC	600	95 (15.9%)	
Basyouni et al. (2020) [55]	BCC	2443	239 (9.8%)	
Brokmeier et al. (2024) [46]	‘white skin cancer’ ***	4000	72.8%	women: 78.2%, men: 67.5%, *p* < 0.001
	‘light skin cancer’ ***	4000	60.9%	women: 64.3%, men: 57.5%, *p* < 0.001
	BCC	4000	30.7%	women: 37.9%, men: 23.7%, *p* < 0.001
	SCC	4000	22.6%	women: 26.9%, men: 18.4%, *p* < 0.001
Diehl et al. (2024) [47]	‘white skin cancer’ ***	905	71.0%	
	‘light skin cancer’ ***	905	61.9%	
	BCC	905	29.5%	
	SCC	905	23.0%	

KC: Keratinocyte carcinoma; SCC: Squamous cell carcinoma; BCC: Basal cell carcinoma. * Calculation based on reported data. ** No further details provided. *** Colloquial German terms for KC.

**Table 3:** Identification of KC as a type of skin cancer, ordered by publication date.

Publication	Identified as a Type of Skin Cancer	N Analyzed	*n* (%) Correct	Further Details
Katz, Jernigan (1991) [48]	Which of the following is not a major form of skin cancer? (BCC, SCC, MM, adenoid cell carcinoma)	251	62%	
Suppa et al. (2013) [52]	BCC	1204	191 (15.9%)	
Pollock (2015) [44]	BCC	91	81 (89.01%) *	women: 88%; men: 90%
	SCC	91	56 (61.54%) *	women: 71%; men: 50%
Duarte et al. (2018) [29]	BCC	2159	37%	
	SCC	2159	27%	
Sideris, Thomas (2020) [45]	BCC	179	30.7%	
	SCC	179	20.7%	
Kyprianou et al. (2022) [58]	BCC	596	23 (3.86%) *	
	SCC	596	49 (8.22%) *	

KC: Keratinocyte carcinoma; BCC: Basal cell carcinoma; SCC: Squamous cell carcinoma; MM: Malignant melanoma. * Rounded calculated values.

**Table 4:** Knowledge regarding the prevalence of KC, ordered by publication date.

Publication	Item	N Analyzed	*n* (%) Correct	Further Details
Spradlin et al. (2010) [51]	The most common form of skin cancer is?	492	5.3%	correct answer: BCC
Hobbs et al. (2014) [53]	The most common form of skin cancer is?	343	5.2%	correct answer: BCC
Joseph et al. (2020) [57]	What is the most common form of skin cancer?	75	16%	correct answer: BCC
Brokmeier et al. (2024) [46]	Estimated prevalence of NMSC compared to MM	3953	1081 (27.4%)	correct answer: More prevalent Less prevalent: *n* = 797 (20.2%); Equally prevalent: *n* = 971 (24.6%); I do not know: *n* = 1103 (27.9%)

KC: Keratinocyte carcinoma; NMSC: Nonmelanoma skin cancer; MM: Malignant melanoma; BCC: Basal cell carcinoma.

**Table 5:** Specific knowledge regarding KC, ordered by publication date.

Publication	Item		N Analyzed	*n* (%) Correct	Further Details
Katz, Jernigan (1991) [48]	Which of the following is not a sign of basal cell carcinoma: (a) an open sore that is slow to heal (b) a shiny bump or nodule on the skin, usually the face (c) a reddish patch or irritated area that does not go away (d) a black mole with hair growing in it		251	59% *	Incorrect answers (d) in total: 41%
	Basal cell carcinomas: (a) are rarely found in Caucasians (b) tend to metastasize quickly (c) are the most common and least serious of the skin cancers (d) can be fatal if not treated promptly		251	48% *	Incorrect answers (a,b,d) in total: 52%
	Squamous cell carcinomas: (a) can metastasize and cause death (b) are almost always benign (c) tend to occur more frequently in dark-skinned persons (d) usually begin in a mole		251	14%*	Incorrect answers (b,c,d) in total: 86%
Garcia et al. (2020) [56]	Correct identification of pictures of lesions as malignant ‘cancer’ or benign ‘not cancer’	BCC (Picture A) **	37	49%	
		BCC (Picture B) **	37	43%	
		SCC (Picture A) **	37	73%	
		SCC (Picture B) **	37	67%	
Brokmeier et al. (2024) [46]	What do you think are signs of NMSC?	Reddish, rough, scaly skin spots	3968	72.8%	
		Bleeding or poorly healing skin spots	3970	60.9%	
		Alterations in nevi	3970	30.7%	answering ‘no’ was correct
		Light or white spots on the skin	3967	22.6%	answering ‘no’ was correct
	What do you believe contributes to the development of NMSC?	Sunbathing	3978	78.7%	
		UV radiation during outdoor occupation	3973	77.1%	
		Using tanning beds	3977	73.0%	
		Weakened immune system	3978	57.9%	
	What do you reckon are possible consequences of white skin cancer ***?	Surgery	3967	55.5%	
		Metastatic spread	3954	42.2%	
		Radiation therapy or chemotherapy	3967	45.4%	
		Recurrence of NMSC	3954	56.3%	
		NMSC can progress to MM	3959	14.7%	answering ‘no’ was correct
	Estimated severity of NMSC compared to MM		3963	1148 (29.0%)	correct answer: Less severe; Equally severe 1483 (37.4%) More severe 492 (12.4%) I do not know 839 (21.2%)
Diehl et al. (2024) [47]	What do you think are signs of NMSC?	Reddish, rough, scaly skin spots	905	40.7%	
		Bleeding or poorly healing skin spots	905	41.7%	
		Alterations in nevi	905	23.2%	answering ‘no’ was correct
		Light or white spots on the skin	905	18.0%	answering ‘no’ was correct
	Sunlight can contribute to the development of ‘white skin cancer’ *** in people who work outdoors.		905	71.4%	

KC: Keratinocyte carcinoma; NMSC: Nonmelanoma skin cancer; MM: Malignant melanoma; BCC: Basal cell carcinoma. * Calculation based on reported data (i.e., 100% incorrect %). ** No further description provided. *** Colloquial German terms for KC.

**Table 6:** Concern about developing KC.

Publication	Item	N analyzed	Results
Brokmeier et al. (2024) [46]	Concern about NMSC	3939	Yes: *n* = 986 (25.0%); No: *n* = 1482 (37.6%); I have never thought about it: *n* = 1471 (37.3%)
Diehl et al. (2024) [47]	Concern about NMSC	905	Yes: 30.0%; No: 38.0%; I have never thought about it: 32.0%

KC: Keratinocyte carcinoma; NMSC: Nonmelanoma skin cancer.

## Data Availability

The original contributions presented in this study are included in the article/supplementary material. Further inquiries can be directed to the corresponding author.

## Supplementary Materials

The following supporting information can be downloaded at: https://www.mdpi.com/article/10.3390/healthcare13151912/s1. Table S1: PRISMA 2020 Checklist; Text S1: Search string by Database; Table S2: List of excluded studies at full-text screening stage by exclusion reason.

## Abbreviations

The following abbreviations are used in this manuscript:

KC	Keratinocyte carcinoma
BCC	Basal cell carcinoma
SCC	Squamous cell carcinoma
UV	Ultraviolet
ROB	Risk of Bias
CI	Confidence interval
MM	Malignant melanoma
NMSC	Nonmelanoma skin cancer
k	Number of studies included in the analyses

## References

[1] Majid U., Wasim A., Bakshi S., Truong J. (2020). Knowledge, (mis-)conceptions, risk perception, and behavior change during pandemics: A scoping review of 149 studies. Public Underst. Sci..

[2] Sheeran P., Harris P.R., Epton T. (2014). Does heightening risk appraisals change people’s intentions and behavior? A meta-analysis of experimental studies. Psychol. Bull..

[3] Anand P., Kunnumakkara A.B., Sundaram C., Harikumar K.B., Tharakan S.T., Lai O.S., Sung B., Aggarwal B.B. (2008). Cancer is a preventable disease that requires major lifestyle changes. Pharm. Res..

[4] Lomas A., Leonardi-Bee J., Bath-Hextall F. (2012). A systematic review of worldwide incidence of nonmelanoma skin cancer. Br. J. Dermatol..

[5] Leiter U., Keim U., Garbe C., Reichrath J. (2020). Epidemiology of Skin Cancer: Update 2019. Sunlight, Vitamin D and Skin Cancer.

[6] Brochez L., Volkmer B., Hoorens I., Garbe C., Röcken M., Schüz J., Whiteman D.C., Autier P., Greinert R., Boonen B. (2025). Skin cancer in Europe today and challenges for tomorrow. J. Eur. Acad. Dermatol. Venereol..

[7] Karimkhani C., Boyers L.N., Dellavalle R.P., Weinstock M.A. (2015). It’s time for “keratinocyte carcinoma” to replace the term “nonmelanoma skin cancer”. J. Am. Acad. Dermatol..

[8] Ferlay J., Ervik M., Lam F., Laversanne M., Colombet M., Mery L., Piñeros M., Znaor A., Soerjomataram I., Bray F. (2024). Global Cancer Observatory: Cancer Today.

[9] Verkouteren J.A.C., Ramdas K.H.R., Wakkee M., Nijsten T. (2017). Epidemiology of basal cell carcinoma: Scholarly review. Br. J. Dermatol..

[10] Green A.C., Olsen C.M. (2017). Cutaneous squamous cell carcinoma: An epidemiological review. Br. J. Dermatol..

[11] Geller A.C., Swetter S.M. (2012). Reporting and registering nonmelanoma skin cancers: A compelling public health need. Br. J. Dermatol..

[12] Global Burden of Disease Collaborative Network (2025). Global Burden of Disease Study 2021 (GBD 2021).

[13] Zhou L., Zhong Y., Han L., Xie Y., Wan M. (2025). Global, regional, and national trends in the burden of melanoma and non-melanoma skin cancer: Insights from the global burden of disease study 1990–2021. Sci. Rep..

[14] Yang D.D., Borsky K., Jani C., Crowley C., Rodrigues J.N., Matin R.N., Marshall D.C., Salciccioli J.D., Shalhoub J., Goodall R. (2023). Trends in keratinocyte skin cancer incidence, mortality and burden of disease in 33 countries between 1990 and 2017. Br. J. Dermatol..

[15] Nanz L., Keim U., Katalinic A., Meyer T., Garbe C., Leiter U. (2024). Epidemiology of Keratinocyte Skin Cancer with a Focus on Cutaneous Squamous Cell Carcinoma. Cancers.

[16] Lear W., Dahlke E., Murray C.A. (2007). Basal cell carcinoma: Review of epidemiology, pathogenesis, and associated risk factors. J. Cutan. Med. Surg..

[17] Chang R.C., Yen H., Heskett K.M., Yen H. (2024). The Role of Health Literacy in Skin Cancer Preventative Behavior and Implications for Intervention: A Systematic Review. J. Prev..

[18] Gordon L.G., Rowell D. (2015). Health system costs of skin cancer and cost-effectiveness of skin cancer prevention and screening: A systematic review. Eur. J. Cancer Prev..

[19] Pil L., Hoorens I., Vossaert K., Kruse V., Tromme I., Speybroeck N., Brochez L., Annemans L. (2016). Burden of skin cancer in Belgium and cost-effectiveness of primary prevention by reducing ultraviolet exposure. Prev. Med..

[20] Holden C.E., Wheelwright S., Harle A., Wagland R. (2021). The role of health literacy in cancer care: A mixed studies systematic review. PLoS ONE.

[21] Kelly P.A., Haidet P. (2007). Physician overestimation of patient literacy: A potential source of health care disparities. Patient Educ. Couns..

[22] Nahar V.K., Wilkerson A.H., Pearlman R.L., Ferris T.S., Zardoost P., Payson S.N., Aman I., Quadri S.S.A., Brodell R.T. (2020). Skin cancer-related knowledge, attitudes, beliefs, and practices among the population in Gulf Cooperation Council countries: A systematic search and literature review. Arch. Dermatol. Res..

[23] Fernandez-Ruiz J., Montero-Vilchez T., Buendia-Eisman A., Arias-Santiago S. (2022). Knowledge, Behaviour and Attitudes Related to Sun Exposure in Sportspeople: A Systematic Review. Int. J. Environ. Res. Public Health.

[24] Ziehfreund S., Schuster B., Zink A. (2019). Primary prevention of keratinocyte carcinoma among outdoor workers, the general population and medical professionals: A systematic review updated for 2019. J. Eur. Acad. Dermatol. Venereol..

[25] Page M.J., McKenzie J.E., Bossuyt P.M., Boutron I., Hoffmann T.C., Mulrow C.D., Shamseer L., Tetzlaff J.M., Akl E.A., Brennan S.E. (2021). The PRISMA 2020 statement: An updated guideline for reporting systematic reviews. Syst. Rev..

[26] Cooke A., Smith D., Booth A. (2012). Beyond PICO: The SPIDER tool for qualitative evidence synthesis. Qual. Health Res..

[27] Wohlin C. Guidelines for snowballing in systematic literature studies and a replication in software engineering. Proceedings of the 18th International Conference on Evaluation and Assessment in Software Engineering.

[28] Haddaway N.R., Grainger M.J., Gray C.T. (2022). Citationchaser: A tool for transparent and efficient forward and backward citation chasing in systematic searching. Res. Synth. Methods.

[29] Duarte A.F., Nagore E., Silva J.N.M., Picoto A., Pereira A.C., Correia O.J.C. (2018). Sun protection behaviour and skin cancer literacy among outdoor runners. Eur. J. Dermatol..

[30] MacKie R.M. (2004). Awareness, knowledge and attitudes to basal cell carcinoma and actinic keratoses among the general public within Europe. J. Eur. Acad. Dermatol. Venereol..

[31] Miles A., Waller J., Hiom S., Swanston D. (2005). SunSmart? Skin cancer knowledge and preventive behaviour in a British population representative sample. Health Educ. Res..

[32] Pfahlberg A., Gefeller O., Kolmel K.F. (1997). Public awareness of malignant melanoma risk factors in Germany. J. Epidemiol. Community Health.

[33] McGowan J., Sampson M., Salzwedel D.M., Cogo E., Foerster V., Lefebvre C. (2016). PRESS Peer Review of Electronic Search Strategies: 2015 Guideline Statement. J. Clin. Epidemiol..

[34] The EndNote Team (2013). EndNote.

[35] Ouzzani M., Hammady H., Fedorowicz Z., Elmagarmid A. (2016). Rayyan-a web and mobile app for systematic reviews. Syst. Rev..

[36] Munn Z., Moola S., Lisy K., Riitano D., Tufanaru C. (2015). Methodological guidance for systematic reviews of observational epidemiological studies reporting prevalence and cumulative incidence data. Int. J. Evid. Based Healthc..

[37] Higgins J.P., Thompson S.G., Deeks J.J., Altman D.G. (2003). Measuring inconsistency in meta-analyses. BMJ.

[38] Higgins J.P.T., Thompson S.G., Spiegelhalter D.J. (2009). A Re-Evaluation of Random-Effects Meta-Analysis. J. R. Stat. Soc. Ser. A Stat. Soc..

[39] Guddat C., Grouven U., Bender R., Skipka G. (2012). A note on the graphical presentation of prediction intervals in random-effects meta-analyses. Syst. Rev..

[40] Barker T.H., Migliavaca C.B., Stein C., Colpani V., Falavigna M., Aromataris E., Munn Z. (2021). Conducting proportional meta-analysis in different types of systematic reviews: A guide for synthesisers of evidence. BMC Med. Res. Methodol..

[41] Zink A., Koch E., Seifert F., Rotter M., Spinner C.D., Biedermann T. (2016). Nonmelanoma skin cancer in mountain guides: High prevalence and lack of awareness warrant development of evidence-based prevention tools. Swiss Med. Wkly..

[42] Zink A., Thome F., Schielein M., Spinner C.D., Biedermann T., Tizek L. (2018). Primary and secondary prevention of skin cancer in mountain guides: Attitude and motivation for or against participation. J. Eur. Acad. Dermatol. Venereol..

[43] Zink A., Wurstbauer D., Rotter M., Wildner M., Biedermann T. (2017). Do outdoor workers know their risk of NMSC? Perceptions, beliefs and preventive behaviour among farmers, roofers and gardeners. J. Eur. Acad. Dermatol. Venereol..

[44] Pollock C. (2015). Skin cancer awareness in the Northern Rivers: The gender divide. Aust. Med. Stud. J..

[45] Sideris E., Thomas S.J. (2020). Patients’ sun practices, perceptions of skin cancer and their risk of skin cancer in rural Australia. Health Promot. J. Aust..

[46] Brokmeier L.L., Görig T., Spähn B.A., Breitbart E.W., Heppt M., Diehl K. (2024). What does the general population know about nonmelanoma skin cancer? Representative cross-sectional data from Germany. J. Public Health.

[47] Diehl K., Dursun E., Görig T. (2024). Berufskrankheit UV-induzierter Hautkrebs. Zentralblatt Arbeitsmedizin Arbeitsschutz Ergon..

[48] Katz R.C., Jernigan S. (1991). Brief report: An empirically derived educational program for detecting and preventing skin cancer. J. Behav. Med..

[49] Halpern A.C., Kopp L.J. (2005). Awareness, knowledge and attitudes to non-melanoma skin cancer and actinic keratosis among the general public. Int. J. Dermatol..

[50] Ashinoff R., Levine V.J., Steuer A.B., Sedwick C. (2009). Teens and tanning knowledge and attitudes. J. Clin. Aesthet. Dermatol..

[51] Spradlin K., Bass M., Hyman W., Keathley R. (2010). Skin cancer: Knowledge, behaviors, and attitudes of college students. South. Med. J..

[52] Suppa M., Cazzaniga S., Fargnoli M.C., Naldi L., Peris K. (2013). Knowledge, perceptions and behaviours about skin cancer and sun protection among secondary school students from Central Italy. J. Eur. Acad. Dermatol. Venereol..

[53] Hobbs C., Nahar V.K., Ford M.A., Bass M.A., Brodell R.T. (2014). Skin cancer knowledge, attitudes, and behaviors in collegiate athletes. J. Skin. Cancer.

[54] Anders M.P., Nolte S., Waldmann A., Capellaro M., Volkmer B., Greinert R., Breitbart E.W. (2015). The German SCREEN project—Design and evaluation of the communication strategy. Eur. J. Public Health.

[55] Basyouni R.N., Alshamrani H.M., Al-Faqih S.O., Alnajjar S.F., Alghamdi F.A. (2020). Awareness, knowledge, and attitude toward nonmelanoma skin cancer and actinic keratosis among the general population of western Saudi Arabia. J. Family Med. Prim. Care.

[56] Garcia D., Jefferson I.S., Ramirez P., Palomino A., Adams W., Vera J., De La Torre R., Lee K., Elsensohn A., Kazbour H. (2020). Video Education to Promote Skin Cancer Awareness and Identification in Spanish-speaking Patients. J. Clin. Aesthet. Dermatol..

[57] Joseph A., Kindratt T., Pagels P., Gimpel N. (2020). Knowledge, Attitudes, and Practices Regarding Skin Cancer and Sun Exposure among Homeless Men at a Shelter in Dallas, TX. J. Cancer Educ..

[58] Kyprianou D., Charalambidou I., Famojuro O., Wang H., Su D., Farazi P.A. (2022). Knowledge and Attitudes of Cypriots on Melanoma Prevention: Is there a Public Health Concern?. BMC Public Health.

[59] Roky A.H., Islam M.M., Ahasan A.M.F., Mostaq S., Mahmud Z., Amin M.N., Mahmud A. (2025). Overview of skin cancer types and prevalence rates across continents. Cancer Pathog. Ther..

[60] Bray F., Jemal A., Torre L.A., Forman D., Vineis P. (2015). Long-Term Realism and Cost-Effectiveness: Primary Prevention in Combatting Cancer and Associated Inequalities Worldwide. JNCI J. Natl. Cancer Inst..

[61] Lee H.Y., Lee J., Kim N.K. (2015). Gender Differences in Health Literacy Among Korean Adults: Do Women Have a Higher Level of Health Literacy Than Men?. Am. J. Mens. Health.

[62] Oliffe J.L., Rossnagel E., Kelly M.T., Bottorff J.L., Seaton C., Darroch F. (2020). Men’s health literacy: A review and recommendations. Health Promot. Int..

[63] Rasheed N. (2024). Melanoma awareness programs and their impact on the life of Australian Queenslanders: A concise analysis. Int. J. Health Sci..

[64] Garbe C., Keim U., Gandini S., Amaral T., Katalinic A., Hollezcek B., Martus P., Flatz L., Leiter U., Whiteman D. (2021). Epidemiology of cutaneous melanoma and keratinocyte cancer in white populations 1943–2036. Eur. J. Cancer.

[65] Savoye I., Olsen C.M., Whiteman D.C., Bijon A., Wald L., Dartois L., Clavel-Chapelon F., Boutron-Ruault M.-C., Kvaskoff M. (2018). Patterns of Ultraviolet Radiation Exposure and Skin Cancer Risk: The E3N-SunExp Study. J. Epidemiol..

[66] Schapira M.M., Swartz S., Ganschow P.S., Jacobs E.A., Neuner J.M., Walker C.M., Fletcher K.E. (2017). Tailoring Educational and Behavioral Interventions to Level of Health Literacy: A Systematic Review. MDM Policy Pract..

[67] Schmitt J., Seidler A., Diepgen T.L., Bauer A. (2011). Occupational ultraviolet light exposure increases the risk for the development of cutaneous squamous cell carcinoma: A systematic review and meta-analysis. Br. J. Dermatol..

[68] (2021). German Guideline Program in Oncology (German Cancer Society G.C.A., AWMF). Evidence-based Guideline on Prevention of Skin Cancer Long Version 2.1. https://www.leitlinienprogramm-onkologie.de/leitlinien/hautkrebs-praevention/.

[69] Uter W., Eversbusch C., Gefeller O., Pfahlberg A. (2021). Quality of Information for Skin Cancer Prevention: A Quantitative Evaluation of Internet Offerings. Healthcare.

[70] Michie S., van Stralen M.M., West R. (2011). The behaviour change wheel: A new method for characterising and designing behaviour change interventions. Implement. Sci..

[71] Rosenstock I.M. (1974). The Health Belief Model and Preventive Health Behavior. Health Educ. Monogr..

[72] Schwarzer R. (2008). Modeling Health Behavior Change: How to Predict and Modify the Adoption and Maintenance of Health Behaviors. Appl. Psychol..

[73] Ferrer R., Klein W.M. (2015). Risk perceptions and health behavior. Curr. Opin. Psychol..

[74] Paalosalo-Harris K., Skirton H. (2017). Mixed method systematic review: The relationship between breast cancer risk perception and health-protective behaviour in women with family history of breast cancer. J. Adv. Nurs..

[75] Edmonds K.A., Rose J.P., Aspiras O.G., Kumar M.S. (2022). Absolute and comparative risk assessments: Evidence for the utility of incorporating internal comparisons into models of risk perception. Psychol. Health.

[76] Olsen C.M., Pandeya N., Green A.C., Ragaini B.S., Venn A.J., Whiteman D.C. (2022). Keratinocyte cancer incidence in Australia: A review of population-based incidence trends and estimates of lifetime risk. Public Health Res. Pract..

[77] Brokmeier L.L., Diehl K., Spähn B.A., Jansen C., Konkel T., Uter W., Görig T. (2023). “Well, to Be Honest, I Don’t Have an Idea of What It Might Be”—A Qualitative Study on Knowledge and Awareness Regarding Nonmelanoma Skin Cancer. Curr. Oncol..

[78] Zink A., Schielein M., Wildner M., Rehfuess E.A. (2019). ‘Try to make good hay in the shade—It won’t work!’ A qualitative interview study on the perspectives of Bavarian farmers regarding primary prevention of skin cancer. Br. J. Dermatol..

[79] Haney M.O., Bahar Z., Beser A., Arkan G., Cengiz B. (2018). Psychometric Testing of the Turkish Version of the Skin Cancer and Sun Knowledge Scale in Nursing Students. J. Cancer Educ..

[80] Day A.K., Wilson C., Roberts R.M., Hutchinson A.D. (2014). The Skin Cancer and Sun Knowledge (SCSK) Scale: Validity, Reliability, and Relationship to Sun-Related Behaviors Among Young Western Adults. Health Educ. Behav..

[81] von Elm E., Altman D.G., Egger M., Pocock S.J., Gøtzsche P.C., Vandenbroucke J.P. (2007). The Strengthening the Reporting of Observational Studies in Epidemiology (STROBE) statement: Guidelines for reporting observational studies. Lancet.

[82] Gefeller O., Uter W., Pfahlberg A.B. (2016). Long-term development of parental knowledge about skin cancer risks in Germany: Has it changed for the better?. Prev. Med..

